# Etiologies, Risk Factors and Impact of Severe Diarrhea in the Under-Fives in Moramanga and Antananarivo, Madagascar

**DOI:** 10.1371/journal.pone.0158862

**Published:** 2016-07-13

**Authors:** Rindra Vatosoa Randremanana, Richter Razafindratsimandresy, Todisoa Andriatahina, Arthur Randriamanantena, Lovaniaina Ravelomanana, Frédérique Randrianirina, Vincent Richard

**Affiliations:** 1 Epidemiology Unit, Institut Pasteur de Madagascar, Antananarivo, Madagascar; 2 Virology Unit, Institut Pasteur de Madagascar, Antananarivo, Madagascar; 3 Service de Pédiatrie, Centre Hospitalier de District Niveau 2, Moramanga, Madagascar; 4 Centre Hospitalier Universitaire Mère Enfant d’Ambohimiandra, Antananarivo, Madagascar; 5 Clinical Biology Centre, Institut Pasteur de Madagascar, Antananarivo, Madagascar; 6 Epidemiology Unit, Institut Pasteur de Dakar, Dakar, Sénégal; Chang Gung Memorial Hospital, TAIWAN

## Abstract

**Background:**

Diarrheal disease remains a leading cause of death in children in low-income countries. We investigated the etiology, risk factors and effects on nutritional status of severe diarrhea in children from two districts in Madagascar.

**Methods:**

We performed a matched case-control study in 2011 to 2014, on children under the age of five years from Moramanga and Antananarivo. The cases were children hospitalized for severe diarrhea and the controls were children without diarrhea selected at random from the community. Stool samples were collected from both groups. Anthropometric measurements were made during follow-up visits about one and two months after enrolment.

**Results:**

We enrolled 199 cases and 199 controls. Rotavirus infection was the most frequently detected cause of diarrhea. It was strongly associated with severe diarrhea (OR: 58.3; 95% CI: 7.7–439.9), accounting for 42.4% (95% CI: 37.6–43.1) of severe diarrhea cases. At the household level, possession of cattle (OR = 0.3; 95% CI: 0.1–0.6) and living in a house with electricity (OR = 0.4; 95% CI: 0.2–0.8) were protective factors. The presence of garbage around the house was a risk factor for severe diarrhea (OR = 3.2; 95% CI: 1.9–5.4). We found no significant association between severe diarrhea and the nutritional status of the children at follow-up visits, but evident wasting at enrolment was associated with a higher risk of severe diarrhea (OR = 9; 95% CI: 4.5–17.9).

**Conclusions:**

Severe childhood diarrhea is mostly caused by rotavirus infection. An anti-rotavirus vaccine has already been introduced in Madagascar and should be promoted more widely. However, post-licensing surveillance is required. Interventions to improve the nutritional status of children, preventive measures focused on household and personal hygiene and nutritional rehabilitation during severe diarrheal disease should be reinforced.

## Introduction

In 2010, diarrheal diseases were the second most frequent killer of children under the age of five years worldwide. Diarrhea caused 0.7 million deaths among children from this age group[1] and 78% of all pediatric diarrhea-associated deaths occurred in Africa and South-East Asia [2]. In 2000–2013, diarrheal mortality decreased by 6.5% per year [3], with the largest absolute decreases observed in sub-Saharan Africa and Southern Asia. Efforts to decrease the impact of diarrhea require information about its etiology, risk factors and effects, particularly in developing countries, such as Madagascar, in which such data are generally scarce. In Madagascar, a community-based study on the etiology of acute diarrhea was conducted in 2008–2009 [4]. However, only limited information is available concerning the causal agents of severe diarrhea. Inadequate laboratory facilities have resulted in most cases of diarrhea being treated on a symptomatic basis. This may have led to an overestimation of the number of cases of potentially bacterial diarrhea and the misuse of antibiotics in a context of limited knowledge about levels of antibiotic resistance. We tried to bridge these gaps by generating information useful for prevention and control programs through a matched case-control study on severe diarrhea in children under the age of five years. We analyzed the etiology, risk factors and effects on nutritional status of severe diarrhea in children from Antananarivo and Moramanga.

## Methods

### Study Design and Enrolment

This case-control study was conducted in Antananarivo and Moramanga, from November 2011 to January 2014. The cases were children aged 0 to 59 months presenting with severe diarrhea at the Pediatrics Department of *Centre Hospitalier de District Niveau 2* (CHD2) in Moramanga and at the *Centre Hospitalier Universitaire Mère Enfant d'Ambohimiandra* (CHUMEA) in Antananarivo. Both these hospitals are public facilities run by the Ministry of Health of Madagascar and providing services for their local communities. The pediatric ward of CHUMEA has 27 beds and is located in Antananarivo, the capital city of Madagascar. The pediatric ward of CHD2 in Moramanga, in the central eastern region of Madagascar, has 10 beds and was established in 2011. Diarrhea was defined as three or more loose stools in the last 24 h. The inclusion criteria for cases were an acute diarrheal episode (onset within the last seven days), and at least one of the following criteria for severe diarrhea: signs of dehydration (sunken eyes, confirmed by the parent or guardian as being more sunken than usual; loss of skin turgor, detected as abdominal skin pinches with slow [about 2 s] or very slow[>2 s] recovery), and the administration or prescription of intravenous rehydration. We recruited a control for each case enrolled, during home visits. The controls were matched with the cases for age (±2 months for patients aged 0–23 months and ±4 months for patients aged 24–59 months), sex and place of residence (living in the same village as the patient with diarrhea, or in a neighboring village). Controls were enrolled within 14 days of the corresponding case and were randomly selected from the Health and Demographic Surveillance Site of Moramanga (HDSS) database, or from names proposed by community health workers in Antananarivo. Controls were considered eligible for inclusion if they were in good health and had not suffered from diarrhea or used antibiotics in the seven days before the survey.

The protocol was approved by the National Ethics Committee of the Ministry of Public Health of Madagascar (No. 069-MSANP/CE 14/12/2010) and the Clinical Research Committee of the Pasteur Institute (No. 2011–27 25/02/2013). Written informed consent was obtained from the parent or primary carer of each child before the initiation of study activities.

### Specimen and Data Collection

At enrolment, demographic and household characteristics were recorded on questionnaires, for both patients and controls. For cases, information about the history of clinical symptoms, such as fever, vomiting, coughing and the possible use of oral rehydration salts (ORS) before coming to the hospital was collected. A detailed physical examination was conducted by a clinical officer at the time of enrolment, to determine the nutritional status of the cases. Field workers trained in standardized anthropometry determined the weight and length/height of the controls at enrolment. They were also responsible for recruiting controls from the community and for carrying out two follow-up visits to each household, about 30 and 60 days after enrolment. During these follow-up visits, they assessed the child's vital status, asked about the medical events that had occurred between visits and repeated anthropometric measurements.

Fresh stools were collected from all cases and controls. For all the participants in Antananarivo and for controls in Moramanga, fecal specimens were transported within two hours of passage, in cool boxes, to the Clinical Biology Center of the *Institut Pasteur de Madagascar* (CBC-IPM) in Antananarivo or the Hospital Laboratory in Moramanga. Samples from cases hospitalized in the Pediatrics Department of CHD2 in Moramanga were aliquoted and used immediately to inoculate different types of agar medium in the hospital laboratory. The agar plates were then sent to the CBC-IPM for processing.

### Laboratory Procedures

Stools were examined for bacteria, viruses and parasites. The eggs and trophozoites of parasitic agents were detected by microscopy. Bacteria, such as *Salmonella* spp., *Shigella* spp., *Campylobacter* spp. and *Escherichia coli (E*. *coli*), were isolated by conventional culture techniques. Stool samples were plated on Hektoen agar for the detection of *Salmonella* spp. and *Shigella* spp., on eosin methylene blue agar and UriSelect for the detection of *E*. *coli* and on Karmali agar for the detection of *Campylobacter* spp. All media were incubated at 37°C, with specific microaerophilic conditions for *Campylobacter* spp. (‘Campygen’, Oxoid England). *Campylobacter* spp. identification was confirmed with a hemagglutination test kit (Campy dry spot, from Oxoid, UK) performed as recommended by the manufacturer. Previously reported polymerase chain reaction (PCR) methods were used to screen isolates of *E*. *coli* for genes encoding virulence factors associated with diarrheagenic *E*. *coli* (DEC) [5]. For the detection of rotavirus, adenovirus and astrovirus, two aliquots of a fresh stool specimen from each child were kept frozen at –80°C in cryovials, which were sent to the IPM for viral analysis and biobanking. Viral antigen testing was carried out with the commercial Prospect Rotavirus, Prospect Adenovirus, and Prospect Astrovirus immunoassays (Prospect, Oxoid, UK), performed according to the manufacturer’s instructions. The rotavirus-positive samples were then characterized by genotyping, as described by Razafindratsimandresy *et al*. [6].

### Statistical Analysis

We used R software [7] for data analysis. For cases, we used logistic regression analysis to identify the symptoms associated with each of the pathogens detected.

We assessed the association of severe diarrhea with potential pathogens, in conditional logistic regression analyses. We used matched odds ratios and pathogen prevalence among patients with severe diarrhea to calculate the adjusted population attributable fraction (AF), to determine the pathogen-specific diarrhea burden. The adjusted AF was calculated from a multiple conditional logistic regression model including other pathogens significantly associated with severe diarrhea.

Bivariate and multivariate associations between independent variables and the occurrence of severe diarrhea were investigated by conditional logistic regression analyses. The independent variables were the child's nutritional status at enrolment, household characteristics (types of wall /roof/floor, presence of a bathroom/kitchen/electricity, possession of poultry/ cattle), household hygiene (type of toilet facilities, source of drinking water, presence of a bathroom, and presence of garbage in and around the house). Factors identified as predictive, with a *p* value less than 0.2 in bivariate analysis, were included in the multivariate analysis. The final model, which included variables significant with an alpha risk of 0.05, was determined by backward elimination.

We investigated whether severe diarrhea was associated with impaired growth, using length/height-for-age (HAZ) and weight-for-length/height (WHZ) scores. These scores were calculated with WHO Anthro software [8]. According to WHO criteria [9], children were considered to present stunting (HAZ) or wasting (WHZ) if their scores were more than two standard deviations below the mean value for age (SDS of -2 or lower). We calculated HAZ and WHZ scores at enrolment and monitored the changes in these scores from enrolment to follow-up. For this monitoring, we used the ratio of the difference in z-scores between enrolment and follow-up to the z-score at enrolment (ΔWHZ/WHZ_0_ and ΔHAZ/HAZ_0_). Severe dehydration is often associated with weight change, and, as there were no body weight records before the onset of diarrhea, we used estimated pre-diarrhea body weight at enrolment for ΔWHZ calculation [10]. We compared HAZ and WHZ scores at enrolment for all children with severe diarrhea, in paired *t* tests. We compared ΔHAZ/HAZ_0_ and ΔWHZ/WHZ_0_ for all possible matched pairs, in linear regression models, adjusting for HAZ and WHZ scores at enrolment and the duration of follow-up. All statistical analyses were performed with a significance threshold of p value of 0.05.

## Results

During the study, 210 children met the criteria for enrolment as cases and were invited to participate. The final analysis was performed on 199 (94.7%) of these cases: four (1.9%) patients refused to participate and the other seven (3.3%) not included had no matched control. We enrolled 199 matched controls. Table 1 shows the demographic and household characteristics of the cases and their matched controls.

**Table 1:** Individual and household characteristics of the cases and controls from Antananarivo and Moramanga, 2011–2014.

Characteristics	Cases	Controls	*p*-value*
**Individual characteristics**	*n* (%)	*n* (%)	
	199	199	
Mean age in months (SD)	15.6 (10.2)	15.9 (10.1)	-
Female individuals	70	70	-
Breastfed			4 x 10^−3^
Yes	141 (70.9)	166 (83.4)	
No	58 (29.1)	33 (16.6)	
Stunting			NS
Yes	68 (34.5)	79 (40.1)	
No	129 (65.5)	118 (59.9)	
Wasting			4 x 10^−10^
Yes	85 (43.1)	13 (6.6)	
No	112 (56.9)	184 (93.4)	
**Household characteristics**	140 to 142	140 to 142	
Walls			NS
Solid materials (cement, etc.)	117 (82.9)	114 (81.4)	
Less solid materials (mud, plants, etc.)	24 (17.1)	26 (18.6)	
Floor conditions			4x10^-3^
Cement, tiles, wood	130 (92.2)	113 (80.1)	
Unpaved floor	11 (7.8)	28 (19.9)	
Roof			NS
Solid materials (corrugated metal, etc.)	133 (94.3)	133 (95.0)	
Plants	8 (5.7)	7 (5.0)	
Source of drinking water			NS
Clean water¶	132 (93.6)	135 (95.7)	
Water not considered clean	9 (6.4)	6 (4.3)	
Electricity			0.1
Yes	86 (61.4)	100 (70.9)	
No	54 (38.6)	41 (29.1)	
Toilets			NS
None	5 (3.5)	4 (2.8)	
Modern	33 (23.4)	27 (19.1)	
Traditional	103 (73.1)	110 (78.1)	
Bathroom			NS
Yes	101 (71.6)	89 (63.1)	
No	40 (28.4)	52 (36.9)	
Cooking area			NS
Yes	110 (78.0)	113 (80.1)	
No	31 (22.0)	28 (19.9)	
Poultry			NS
Yes	40 (28.2)	46 (32.4)	
No	102 (71.8)	96 (67.6)	
Cattle			2 x 10^−3^
Yes	9 (6.3)	27 (19.0)	
No	133 (93.7)	115 (81.0)	
Garbage on site			5x10^-4^
Yes	86 (61.0)	56 (39.7)	
No	55 (39.0)	85 (60.3)	

*: p-value from univariate analysis; SD: standard deviation;

^¶^: clean water is piped drinking water, or water from a public tap, tubewell or covered well; NS: not significant

The median age of the children was 13 months (interquartile range: 9 to 19 months), 42.2% (168/398) were under the age of 12 months and 64.8% (258/398) were male. We found significant difference in sanitation and household living conditions between the children with severe diarrhea and their matched controls. Children living in houses with floors made of solid materials (e.g. cement, or tiles) and those living in houses containing or surrounded with garbage were more likely to have severe diarrhea. Our data also suggested that control families were more likely to have cattle than the families of children with severe diarrhea, and that controls were more likely to be breastfed than children with severe diarrhea. At enrolment, wasting was more frequent in children with diarrhea than in controls.

### Clinical and Microbiological Data

The three main symptoms presented by children with severe diarrhea were vomiting (82.9%), fever ≥ 37.5°C (47.7%) and coughing (45.2%). About 61.8% of children with severe diarrhea had passed more than six stools in the last 24 h, 51% had mucus in their stools and 7.4% had blood in their stools. Many children had taken ORS (43.7%) or antibiotics (38.7%) in the 24 h preceding their arrival at the hospital.

The mean duration of hospitalization was 6 (±26) days. During hospitalization, 38.7% of children received antibiotics, 10.6% received zinc and 6.5% were treated by intravenous rehydration.

In children with diarrhea, the frequency of clinical symptoms (vomiting, fever, and cough) was similar for all types of infection except for rotavirus infection. Rotavirus infection was characterized by higher rates of vomiting than for the other types of infection (OR = 3.5, 95% CI: 1.4–8.6; *p* = 5 x 10^−3^) (Table 2).

**Table 2:** Clinical symptoms associated with the enteric pathogens isolated from children with diarrhea in Antananarivo and Moramanga, 2011–2014.

	Cases detected	Number of positive cases		
Agents		Vomiting	Fever	Cough
		n (%)	n (%)	
*Salmonella* spp.	0	0	0	0
*Shigella* spp.	3	2 (66.7%)	3 (100%)	0 (0%)
*Campylobacter* spp.	2	1 (50.0%)	1 (50.0%)	0 (0%)
*Rotavirus*	86	79 (91.9%)	34 (39.5%)	33 (38.4%)
*Astrovirus*	5	5 (100%)	1 (20.0%)	1 (20.0%)
*Adenovirus*	6	5 (83.3%)	1 (16.7%)	4 (66.7%)
*EPEC*	7	6 (85.7%)	1 (14.3%)	2 (28.6%)

*EPEC:* *enteropathogenic E*. *coli*

Table 2 shows the clinical symptoms associated with the various enteric pathogens isolated from children with diarrhea in this study.

We examined stools from all cases and controls for the presence of bacterial pathogens and parasites, but some samples were too small for viral analysis. A potential pathogen was found in 48.2% (96/199) of cases and 20.6% (41/199) of controls. A virus was found in 46.5% of the cases and 15.6% of the controls tested; a bacterial pathogen was found in 6% of cases and 3.5% of controls. We detected no parasites in the children with diarrhea, but 2.5% of the control children were infected with at least one parasite. The bacteria most frequently isolated were enteropathogenic *E*. *coli (EPEC)*: *EPEC* serogroup I in two children, *EPEC* serogroup II in four, *EPEC* serogroup III in four and *EPEC serogroup IV* in one child. *Shigella* was detected in five children: *Shigella sonnei* in three children (2 cases and 1 control) and *Shigella boydii* in two (1 case and 1 control).

More than one microbe was found in the stool samples of 12 cases (6%) and two controls (1%). Five children were infected with both EPEC and rotavirus, three children with both rotavirus and astrovirus, two children with both rotavirus and *Ascaris* and one child each was infected with the following combinations: *Campylobacter* and rotavirus, *Shigella* and rotavirus, rotavirus and adenovirus, EPEC and astrovirus. We found a statistically significant association between the occurrence of severe diarrhea and mixed infection (crude OR: 6; 95% CI: 1.3–26.8).

Rotavirus was the most frequently detected pathogen and rotavirus infection was the factor most strongly associated with severe diarrhea (OR: 58.3, 95% CI: 7.7–439.9) (Table 3). Overall, 42.4% (95% CI: 37.6–43.1) of severe diarrhea cases at the two sites could be attributed to rotavirus.

**Table 3:** Case-control results for the pathogens isolated in Antananarivo and Moramanga, 2011–2014.

Pathogens		Cases		Controls		
	Tested	Positive	Tested	Positive	Crude OR (95% CI)	Adjusted OR^a^ (95% CI)
		n (%)		n (%)		
**Bacteria**	199		199			
*Salmonella* spp.		0 (0.0)		0 (0.0)	-	-
*Shigella* spp.		3 (1.5)		2 (1.0)	1.5 (0.2–8.9)	4.1 (0.4–41.7)
*Campylobacter* spp.		2 (1.0)		1 (0.5)	-	-
*EPEC*		6 (3.0)		5 (2.5)	1.7 (0.5–6.0)	1.6 (0.2–10.9)
**Viruses**	198		198			
*Rotavirus*		86 (43.4)		19 (9.6)	68.0 (9.4–489.7)	58.3 (7.7–439.9)
*Adenovirus*		6 (3.0)		4 (2.0)	1.5 (0.4–5.3)	2.1 (0.4–9.4)
*Astrovirus*		5 (2.5)		9 (4.5)	0.5 (0.2–1.6)	0.6 (0.2–1.9)
**Parasites**	199		199			
*Ascaris lumbricoides*		0 (0.0)		4 (2.0)	-	-
*Tricocephales*		0 (0.0)		1 (0.5)	-	-

^a^: Matched-adjusted for nutritional status (presence or absence of wasting) and breastfeeding status;

EPEC: enteropathogenic E. coli; n: number

Among the 103 rotavirus sequences for which both G and P types were identified, G1P[8] was the most common (*n* = 37; 36%) followed by G2P[4] (*n* = 32; 31.1%), and G3P[8] (*n* = 8; 7.8%).

### Determinants of Severe Diarrhea

Wasting at enrolment was associated with a higher risk of severe diarrhea (OR = 9; 95% CI: 4.5–17.9); wasting was observed in 43.6% of children with diarrhea but only 8.2% of those without diarrhea.

At the household level, the possession of cattle (OR = 0.3; 95% CI: 0.1–0.6) and living in a house with electricity (OR = 0.4; 95% CI: 0.2–0.8) were protective factors. Children living in houses containing or surrounded by garbage were three times more likely to develop severe diarrhea than those living in garbage-free houses (OR = 3.2; 95% CI: 1.9–5.4). The probability of developing severe diarrhea was 4.3 times higher (95% CI: 2.0–9.6) in children living in houses with floors made of solid materials than in those living in households with unpaved floors.

### Follow-Up

During follow-up visits at about 30 and 60 days after enrolment, five (2.5%) deaths were recorded among the 199 children with severe diarrhea, whereas no deaths had occurred in the controls. Three of these deaths occurred during hospitalization, the other two occurring between hospital discharge and the first follow-up visit. All these deaths occurred in children under the age of 18 months with faltering growth (one child with stunting and four children with wasting at enrolment).

Mean WHZ at enrolment, and the change in WHZ (ΔWHZ/WHZ_0_) during follow-up, at the 30-day and 60-day visits, are summarized for cases and controls in Table 4.

**Table 4:** Comparison of weight-for-height or length (WHZ) scores at enrolment, and change in WHZ (ΔWHZ/WHZ_0_) between enrolment and follow-up at 30 and 60 days, between children with diarrhea and their matched controls from Antananarivo and Moramanga, 2011–2014.

Parameter	Status *n*	Mean value	*p*-value
Enrolment WHZ	Cases 198	-0.3 (-0.6 to -0.03)	10^−14^
	Controls 198	-0.1 (-0.4 to 0.2)	
ΔWHZ/WHZ_0_ at 30 days	Cases 185	-1.5 (-3.5 to 0.4)	NS
	Controls 185	-0.3 (-1.5 to 0.8)	
ΔWHZ/WHZ_0_ at 60 days	Cases 175	-0.6(-1.5 to 0.4)	NS
	Controls 175	-0.2 (-1.1 to 0.8)	

n: number; NS: not significant

Mean HAZ did not differ significantly between cases and controls at enrolment. By contrast, mean WHZ at enrolment was significantly lower in children with severe diarrhea than in controls (*p* = 10^−14^); wasting was observed at enrolment in 43.1% of children with diarrhea but only in 6.6% of the control children without diarrhea. Neither the WHZ nor the HAZ scores of the children changed between enrolment and the follow-up visits at 30 and 60 days, after adjustment for WHZ or HAZ at enrolment and the duration of follow-up.

## Discussion

In this matched case-control study we investigated the etiology and determinants of severe diarrhea and its impact on the nutritional status of children in Moramanga and Antananarivo, Madagascar. Rotavirus was the most frequently isolated pathogen and was significantly associated with severe diarrhea. Our study, conducted before the introduction of rotavirus vaccination supports the decision taken by the Ministry of Public Health in May 2014 to introduce rotavirus vaccination. This intervention may decrease the frequency of severe diarrhea by about 37.6% to 43.1%. Severe diarrhea did not seem to have an effect on the nutritional status of the children at follow-up visits, although children with wasting at enrolment were nine times more likely to have diarrhea than those without wasting. Preventive and therapeutic strategies for improving the nutritional status of children and the algorithm for diarrhea management in children should be reinforced.

Our results confirm that rotavirus is the principal causal agent of pediatric diarrhea in Moramanga and Antananarivo. Rotavirus accounted for more than two fifths of diarrhea cases and 92.4% of the children infected with rotavirus were less than two years old. Our findings are highlight consistent with those for other developing countries, in which rotavirus has been reported to be the leading cause of diarrheal disease [11–15]. A monovalent human rotavirus vaccine was introduced into Madagascar’s national immunization program in May 2014. The recommended vaccination schedule is as follows: first dose at the age of 6 to 16 weeks of age, with a second dose administered four weeks after the first dose. Most (75%) of the rotavirus strains detected in this study were of the most common G1 P[8] genotype, followed by the G2[P4] and G3[P8] genotypes; 39.8% of the rotavirus strains identified did not have a G or P type in common with the vaccine strain used in Madagascar. A number of studies have shown that the human rotavirus vaccine protects against several rotavirus strains [16, 17], but potentially waning immunity may be a concern for diarrhea caused by strains with neither the G or P type of the vaccine strain [18]. Post-licensing monitoring of vaccine efficacy and continual surveillance of the rotavirus strains circulating in Madagascar are therefore essential, to evaluate the potential impact of vaccination on genotype diversity.

Children with severe diarrhea had symptoms of respiratory infection, such as coughing. Coincidental infection with respiratory viruses may have occurred. Alternatively, the respiratory symptoms may have been extra-intestinal symptoms of rotavirus infection, which tends to be systemic rather than restricted to the jejunal mucosa [19].

Our data suggest that severe diarrhea did not affect growth during follow-up, although wasting at enrolment was significantly associated with a higher risk of severe diarrhea. A study in Bangladesh [20] showed that the effects of diarrhea on growth may be dependent on the clinical type of diarrhea, with dysentery having the most severe effects. Dysentery was not frequent in the two pediatric wards; only 7.4% of the children with diarrhea had blood in their stools. Breastfeeding may provide some protection against poor weight gain [20]. Our data suggest that, during their diarrheal episodes, 72.8% of children with feeding problems (complementary food intake impossible or reduced) continued to take breast milk, and the risk of wasting was 69% lower in breastfed children than in non-breastfed children. An association between wasting and the occurrence of diarrhea has been reported before [21, 22]. Malnutrition, including wasting in particular, has been shown to increase diarrhea severity and the risk of death [13]. Four of the five deaths occurred in children presenting with wasting at enrolment. Children with wasting require more careful medical attention during their recovery from severe diarrhea and should be followed for at least the first month after hospitalization, as they are at higher risk of death. The association between wasting and severe diarrhea at enrolment may reflect previous episodes of diarrhea, but it was not possible to establish causal relationships in this study based on a case-control design. Our findings highlight the need to improve strategies to prevent undernutrition and for nutrition rehabilitation during diarrheal episodes.

We found that household hygiene, as assessed by considering the presence or absence of garbage in and around the house, was associated with the risk of severe diarrhea. We also found that living in a house with a floor made from solid materials (e.g. cement, tiles) was associated with a higher risk of severe diarrhea. These findings conflict with those of a previous study carried out elsewhere [23] and with those underlying the recommendations of the World Bank [24] indicating that the replacement of dirt floors with cement floors decreased diarrhea rates by 49%. The higher risk of severe diarrhea in children living in households with ‘improved’ floors in our study may reflect unhygienic behavior by the parents, the child or other members of the household, or generally unhygienic conditions in the household. However, data concerning these aspects were not collected in this study. Well-known preventive strategies, such as maintaining household and personal hygiene, should be promoted. We found that the control families were more likely to have cattle than the families of children with severe diarrhea. Evidence for interspecies transmission and for genetic recombination between human and animal rotaviruses (from calves, piglets, avian species) has been reported in several studies [25–27]. These processes appear to contribute to the genetic diversity found in human rotaviruses and may play a role in low efficacy of vaccination in low-income countries [28]. In our study, the possession of cattle may be a proxy for a high household socio-economic status, which has been shown to have an effect on the occurrence of diarrhea [29].

Noroviruses are now recognized as the leading cause of acute gastroenteritis worldwide, responsible for 18% of all acute gastroenteritis cases [30]. We were unable to test for this virus due to financial constraints, but a previous study in Antananarivo showed that only 6% of children with acute diarrhea were infected with norovirus [31]. We faced recruitment problems in this study, because the incidence of severe diarrhea was lower than expected. We were able to include only one third the number of patients initially planned. Mean inclusion rates were four to five cases of severe diarrhea per month at Moramanga and eight cases per month at Antananarivo. Severe diarrhea was not common in the pediatric populations studied, by contrast to the GEMS study [14], which reported an incidence of moderate-to-severe diarrhea of 20 episodes/100 child-years. A cohort study in Moramanga [32] revealed the incidence of acute diarrhea to be low, only one quarter that estimated for low-income countries (0.7 episodes per child-year), with only two children hospitalized for diarrhea complicated by dehydration. Lower levels of exposure to pathogens (e.g. drinking water controls in Antananarivo), programs for diarrhea prevention or management in the community, or health facilities more efficient than anticipated may account for the difference between our findings and predictions for developing countries. Community health worker programs, such as integrated community case management, have already been implemented in Moramanga.

In conclusion, rotavirus was the leading cause of severe diarrhea in children under the age of five years in Moramanga and Antananarivo. Rotavirus vaccination had already been carried out for one year in Madagascar at the time of the publication of the study. However, it should be more widely promoted, and post-licensing monitoring of efficacy should be carried out, together with continuous surveillance of the rotavirus strains circulating in Madagascar. In addition, interventions to improve the nutritional status of children, preventive measures focusing on hygiene, and nutrition rehabilitation during severe diarrheal disease should be reinforced.

## Supporting Information

S1 Supporting Information (ZIP)
